# HealthySMS Text Messaging System Adjunct to Adolescent Group Cognitive Behavioral Therapy in the Context of COVID-19 (Let’s Text!): Pilot Feasibility and Acceptability Study

**DOI:** 10.2196/49317

**Published:** 2024-02-19

**Authors:** Lauren M Haack, Courtney C Armstrong, Kate Travis, Adrian Aguilera, Sabrina M Darrow

**Affiliations:** 1 Department of Psychiatry and Behavioral Sciences University of California San Francisco San Francisco, CA United States; 2 School of Social Welfare University of California Berkeley Berkeley, CA United States

**Keywords:** depression, adolescents, evidence-based intervention, texting, SMS text message, cognitive behavioral therapy, CBT, group CBT, shelter-in-place, COVID-19, mobile health, mHealth, therapy, cognitive, behavior, web-based therapy, e-therapy, youth, young adults, mobile phone

## Abstract

**Background:**

The widespread occurrence and devastating impact of adolescent depression warrant health service research focused on feasible and acceptable digital health tools to supplement evidence-based intervention (EBI) efforts, particularly in the context of shelter-in-place guidelines disrupting youth socialization and service use in the wake of the COVID-19 pandemic. Given the promise of SMS text message interventions to enhance EBI engagement, our team developed the HealthySMS system as an adjunct to one of the most empirically supported interventions for adolescent depression: cognitive behavioral therapy (CBT) group services. The system sends daily SMS text messages requesting responses assessing mood, thoughts, and activities; weekly attendance reminder messages; daily tips about adherence (eg, a prompt for activity completion); and personalized responses based on participants’ texts.

**Objective:**

This study aims to evaluate the feasibility and acceptability of HealthySMS in a real-world setting and explore potential mechanisms of change in EBI engagement, before evaluating the system’s impact on adolescents’ group CBT engagement and, ultimately, depression outcomes.

**Methods:**

Over the course of 2020, we invited all 20 adolescents receiving CBT group services for depression at an outpatient psychiatry clinic to enroll in our HealthySMS study; ultimately, 17 (85%) adolescents agreed to participate. We tracked participant initiation and engagement with the HealthySMS system as well as the content of SMS text message responses to HealthySMS. We also invited each participant to engage in a semistructured interview to gather additional qualitative inputs on the system.

**Results:**

All (n=17, 100%) research participants invited agreed to receive HealthySMS messages, and 94% (16/17) of the participants maintained use during the first month without opting out. We uncovered meaningful qualitative themes regarding the feasibility and acceptability of HealthySMS, as well as its potential impact on EBI engagement.

**Conclusions:**

Taken together, the results of this pilot study suggest that HealthySMS adjunct to adolescent CBT group depression services is feasible and acceptable, as evidenced by high rates of HealthySMS initiation and low rates of dropout, as well as meaningful themes uncovered from participants’ qualitative feedback. In addition, the findings provide evidence regarding iterative improvements to the HealthySMS system and research protocol, as well as potential mechanisms of change for enhanced EBI engagement and, ultimately, adolescent depression outcomes, which can be used in future effectiveness research.

## Introduction

### Background

Depression is a major public health concern for adolescents [1-3]; it is a risk factor for mental health problems that occur in adults, medical illnesses, disability, substance abuse, and suicide [2-6]. Evidence-based efforts to improve adolescent depression and related risks became more imperative during the COVID-19 pandemic owing to concerns about adolescents’ mental health deterioration in the context of shelter-in-place (SIP) guidelines causing substantial educational and social disruptions [7-10]. Further, most in-person services were halted during the implementation of SIP guidelines, rapidly necessitating a transition to telehealth and digital health (dHealth) supports [7,11-14]. In response, the need for pilot feasibility and acceptability testing of dHealth tools targeting adolescent depression in real-world settings became glaringly apparent. Such efforts would provide an essential first step toward testing the effectiveness of dHealth tools for adolescent depression. The impact of this work would extend beyond the initial COVID-19 SIP context, given that research suggests that many systems will and should continue using telehealth and dHealth supports in the foreseeable future [13,15,16], which may be particularly beneficial in reducing disparities for historically marginalized populations [15].

### Evidence-Based Intervention for Adolescents With Depression

There are several effective evidence-based interventions (EBIs) for adolescent depression [17-19]. The most well-supported psychosocial approach is cognitive behavioral therapy (CBT [18,19]), which targets unhelpful thinking patterns and the avoidance of goal-directed and social activities, which are characteristic of depression [20-22]. CBT can be effectively delivered via a group format [23], demonstrating efficacy similar to that of individual CBT in decreasing depression in a more cost-effective modality [24]. CBT can also be delivered electronically (electronic CBT [eCBT]), enhancing accessibility by eliminating the requirement for patients to travel for in-person services [25-29]. The potential benefits of electronically delivered EBIs became increasingly evident in the context of the implementation SIP guidelines during the COVID-19 pandemic, which prohibited many individuals from receiving in-person mental health treatment. Importantly, our society may experience future SIP guidelines during COVID-19 surges, safety lockdowns, or natural disasters in response to climate change. Fortunately, recent research supports the effectiveness and efficacy of eCBT in decreasing depression [25-29].

Despite the promising efficacy research supporting CBT as an EBI for adolescent depression, the effect sizes are heterogeneous and low, demonstrating room for improvement [23,30]. Optimal treatment of adolescent depression requires patient engagement, including the initiation of treatment after referral, the attendance of sessions, the completion of homework between sessions, and the continued engagement in treatment (ie, getting the whole “dose” of treatment [31]). Thus, poor initiation, poor adherence, and treatment dropout are barriers to effective services in real-world settings and are known to mediate treatment outcome [31]. A meta-analysis of EBIs concluded that the average treatment dropout rate is approximately 29% and that patients with depression are at a higher risk for dropout [32]. Treatment dropout appears particularly pronounced among youths and in web-based mental health interventions; therefore, efforts to enhance engagement with eCBT among youth populations are especially called for [33].

### SMS Text Message Interventions as Automated dHealth Supports Aiming to Enhance EBI Engagement

There is a strong scientific premise and public health call to develop and evaluate dHealth supports adjunct to EBIs that are easy to implement, efficient, and sustainable. Using SMS text messages is a promising approach aiming to enhance adolescent EBI engagement by making use of a tool that is readily available and widely used by adolescents. The use of mobile phones is ubiquitous among adolescents, and SMS text messages are used at high rates. As of 2015, more than 85% of adolescents across races and ethnicities had access to a cell phone, and 90% of them used cell phones to send SMS text messages [34]. In fact, two-thirds of adolescents reported that they are more likely to use their cell phones to text rather than talk to friends, and a typical adolescent in the United States sends ≥30 SMS text messages each day [34]. Importantly, in mobile health (mHealth) research, youths report high satisfaction and readily engage with technology [35-37]. In addition, digital technology allows for the automation of SMS text messages; thus, an SMS text message intervention leveraging automation should not add to providers’ burden, making it a more sustainable and efficient services intervention.

SMS text messaging interventions have several advantages over other forms of dHealth interventions, such as app-based and website-based interventions, and are particularly well suited to improve engagement. SMS text messages allow for a more equitable delivery of care, given that they can reach anyone with a phone and do not rely on smartphone ownership or internet access, which are affected by socioeconomic, racial, and ethnic disparities [38]. In addition, although dHealth interventions have demonstrated promise, web-based and mobile app–based interventions are subject to more difficulties with engagement, such as problems with adherence and dropout [26,39].

Some dHealth SMS text message interventions were designed as stand-alone supports (eg, those in the works of Aguilera et al [40] and Bendsten et al [41]; MacDougall et al [42] conducted a scoping review of SMS text message–delivered adolescent mental health interventions); however, there may be unique benefits to SMS text message interventions designed to supplement EBIs and enhance treatment engagement. There are many effective examples of adjunctive SMS text message interventions for disease management and health behavior change (eg, smoking cessation and diabetes management [43]), including SMS text message interventions for adolescents [44]. In the emerging literature on SMS text message interventions, adolescents generally react favorably and show good compliance [35,42,45,46]. Pilot research on integrating SMS text messages into individual CBT has also demonstrated encouraging results [47].

Given the promise of SMS text message interventions to enhance EBI engagement, our team developed the HealthySMS system as an adjunct to group CBT depression services. HealthySMS sends customized SMS text messages to participants, inquiring about mood as well as reminding them to attend CBT and practice strategies learned during group treatment. For safety, adolescent SMS text message responses are monitored for keywords or phrases that could indicate whether someone is expressing suicide risk; providers are sent immediate alerts if the system is triggered by an adolescent SMS text message response with these keywords or phrases. Safety keyword triggers are important features of any automated dHealth system implemented in real-world settings to keep participants safe and providers informed; in addition, this feature may be particularly helpful during the implementation of SIP guidelines, given the probable decrease in adolescent safety monitoring from other settings, such as schools. The addition of HealthySMS to CBT for adults with depression in a public sector treatment setting was associated with an increased number of sessions attended and a longer duration of treatment [48]. The mood ratings sent by adults through HealthySMS also predicted attendance [49]. Thus, adding HealthySMS to EBIs for adolescent depression may be a promising change to existing services, targeting increased engagement and, ultimately, improving outcomes.

### This Study

As a first step in the process of implementing and evaluating the HealthySMS system adjunct to the most evidence-based treatment for adolescent depression (ie, CBT), we conducted a feasibility pilot study in an outpatient clinic embedded within an academic medical center’s department of psychiatry and behavioral sciences. Our primary objectives were to investigate the feasibility and acceptability of HealthySMS in a real-world setting and explore potential mechanisms of change in EBI engagement, before evaluating the system’s impact on adolescents’ group CBT engagement and, ultimately, depression outcomes. We also aimed to monitor the HealthySMS safety keyword alert triggers and provider responses to inform system and research protocol adjustments before future HealthySMS research. This multiphase design featuring a preliminary feasibility pilot to inform decisions about future effectiveness testing is aligned with the Medical Research Council framework [50,51], which has been prolifically used in mental health and dHealth intervention research. We predicted the following hypotheses:

1. Hypothesis 1: most adolescents invited to use HealthySMS (ie, ≥75%) would initiate and maintain use without opting out during their group CBT experience.
2. Hypothesis 2: adolescents enrolled in HealthySMS would display high rates of engagement with the SMS text message system (ie, ≥50% response rate).
3. Hypothesis 3: our team would uncover meaningful qualitative themes from participants’ SMS text message responses and semistructured interviews about the feasibility and acceptability of HealthySMS to inform iterative system and research protocol improvements supporting future HealthySMS effectiveness research.
4. Hypothesis 4: our team would uncover meaningful qualitative themes from participants’ SMS text message responses and semistructured interviews about the potential impact of HealthySMS on EBI engagement to inform decisions on which mechanisms of change to evaluate in future HealthySMS effectiveness research.

## Methods

### Participants

Participants were adolescents aged 13 to 18 years. They were recruited from the University of California San Francisco (UCSF) Department of Psychiatry and Behavioral Sciences Child and Adolescent Services clinic between December 2019 and September 2020. Adolescents were eligible to participate if they were enrolled in the clinic’s group CBT for depression running from January 2020 to September 2020.

Of the 20 eligible adolescents who started the group CBT for depression, 17 (85%) agreed to participate in the study. Participant characteristics are listed in Table 1. Briefly, the average age of the participants was 15.4 (SD 1.5) years, and most participants (15/17, 88%) were diagnosed with major depressive disorder. Most participants (12/17, 71%) had engaged in prior mental health care. Moreover, 18% (3/17) of the participants had a history of at least 1 suicide attempt, with most participants (11/17, 65%) reporting suicidal ideation in the past year. A little more than half (9/17, 53%) of the participants engaged in concurrent individual therapy, family therapy, or a combination.

**Table 1:** Participants’ demographic and clinical information (N=17).

			Values
**Demographic information**			
	Age (years), mean (SD)		15.4 (1.5)
	**Gender, n (%)**		
		Woman	8 (47)
		Man	8 (47)
		Transwoman	1 (6)
	**Ethnicity, n (%)**		
		Latinx	7 (41)
		Non-Latinx	10 (59)
	**Race, n (%)**		
		Asian	1 (6)
		Black	1 (6)
		White	10 (59)
		Biracial or multiracial	4 (24)
		Something else (Hispanic or Mexican)	1 (6)
	**Sexual orientation, n (%)**		
		Heterosexual	8 (47)
		Lesbian	1 (6)
		Bisexual	3 (18)
		Gay	1 (6)
		Pansexual	1 (6)
		Not sure or do not care	3 (18)
**Clinical history, n (%)**			
	**Prior therapy experience^a^**		
		Participated in prior outpatient therapy	9 (53)
		Participated in IOP^b^ or PHP^c^	2 (12)
		Psychiatric hospitalization	1 (6)
		Residential treatment facility	2 (12)
		None	5 (29)
	Prior suicide attempt		3 (18)
	**SI^d^ at intake**		
		Denied	6 (35)
		SI in the past week	3 (18)
		SI in the past month	2 (12)
		SI in the past year	6 (35)
	**NSSI^e^ at intake**		
		Denied	10 (59)
		NSSI in the past week	1 (6)
		NSSI in the past month	2 (12)
		NSSI in the past year	4 (24)
	**School problems^a^**		
		None reported	9 (53)
		IEP^f^ or accommodations	6 (35)
		School refusal	3 (18)
	**Major depressive disorder**		
		Met criteria in the past	1 (6)
		Reports some symptoms	1 (6)
		Currently meets criteria	15 (88)
	**Symptoms from comorbid disorders**		
		Generalized anxiety disorder	5 (29)
		Panic disorder	2 (12)
		Social anxiety disorder	1 (6)
		Eating disorder	1 (6)
		ADHD^g^	1 (6)
		None	7 (41)
	**Concurrent therapy^a^**		
		Individual CBT^h^	6 (35)
		Family therapy	4 (24)
		None	8 (47)
	Concurrent medication management		15 (88)

^a^Response options are not mutually exclusive.

^b^IOP: intensive outpatient treatment.

^c^PHP: partial hospitalization program.

^d^SI: suicidal ideation.

^e^NSSI: nonsuicidal self-injury.

^f^IEP: individualized education plan.

^g^ADHD: attention-deficit/hyperactivity disorder.

^h^CBT: cognitive behavioral therapy.

Of note, our research protocol did not make any changes to how the real-world clinic provided services. Adolescents were not incentivized to start treatment, attend sessions, or complete their homework; they only were incentivized for completing additional research tasks (see below). Adolescents in the clinic could be engaged in individual therapy, family therapy, or both while attending group treatment.

### Ethical Considerations

All study procedures were approved by the UCSF Institutional Review Board (reference number 255820). When participants were minors (ie, age <18 years), their parent or legal guardian completed informed consent and they completed assent procedures; 18-year-old participants completed informed consent procedures. As part of these procedures, participants (and their parents or legal guardians, if applicable) were informed that their information would be kept private and housed on a secure UCSF server only accessible to the study team; they were informed that participants would be compensated with a US $30 gift card for attending the interview).

### CBT Group Intervention

The Cognitive Behavioral Therapy for Depression Group for Adolescents (CBT-D) consists of three 4-week modules on thoughts, activities, and people (ie, 12 group sessions in total). It is based on the Building Recovery by Improving Goals, Habits, and Thoughts (BRIGHT) group CBT manual for depression for adults developed by Miranda et al [52], which was subsequently adapted for adolescents with a diagnosis of major depression or persistent depressive disorder. The thought module involves cognitive interventions with a focus on awareness of helpful and harmful thoughts, the activity module focuses on pleasant activities and behavioral activation, and the people module encourages group members to improve relationships and evaluate the impact of positive and negative social relationships on mood. The sessions are structured with an initial homework check-in followed by a didactic discussion on the covered topic, an interactive discussion led by 2 group providers, and the setting of homework goals related to skill use. In this study, the first 7 group sessions were held in person; the remainder were held via the Health Insurance Portability and Accountability Act–compliant Zoom (Zoom Video Communications, Inc) platform after SIP orders were implemented in March 2020.

### The HealthySMS System

We used the Health Insurance Portability and Accountability Act–compliant, web-based texting platform called HealthySMS, developed by our team member (AA), to send and receive SMS text messages, administer weekly surveys, and track attendance. HealthySMS sent four types of automated SMS text messages to participants: (1) daily mood prompts asking participants to rate their mood and reflect on their mood, thoughts, and behavior (“[First name], what is your mood right now on a scale of 1 to 9 (9 being best)? Please respond with a number and a message about what you are doing or thinking”); (2) personalized and reinforcing responses to 20% of participants’ mood ratings (ie, encouraging the participants to engage in behavioral activation in response to a low mood rating and reinforcing the participants in response to a high mood rating); (3) daily reminders about the concepts and skills learned during the corresponding CBT module for that month; and (4) weekly reminders to attend and come prepared to CBT sent the day before the group session. The participants could opt out of receiving SMS text messages at any point by texting “stop.”

Our clinical research team oriented adolescent participants to the HealthySMS system. We also oriented providers to the SMS text messages and HealthySMS web-based provider dashboard, which visualizes client responses to the SMS text messages, including graphs of mood ratings. HealthySMS monitors participants’ SMS text message responses and alerts providers to words and phrases that may correspond to suicidal behaviors (eg, “die,” “kill,” and “cut”), and providers were trained on how to respond to these alerts in alignment with existing clinic policies and procedures.

### Measures

#### Participants’ Demographics and Clinical History

The participants were asked to complete a demographic survey before beginning group CBT. CBT-D providers recorded psychiatric diagnoses and treatment history information for each participant, including other therapy or medication management services, based on their evaluation and review of the medical record.

#### HealthySMS Engagement

We tracked the number of participants who were invited to receive HealthySMS messages, as well as those who agreed to initiate receipt of HealthySMS messages. We also tracked the number of participants who texted “stop” to opt out of SMS text messages before ending participation in the group, as well as how long each participant received the SMS text messages before opting out. We measured engagement with the HealthySMS messages by tracking the number of mood ratings that participants texted in response to mood rating request messages.

#### HealthySMS Safety Keyword Triggers

We tracked the number and content of participant SMS text message responses that were flagged for the risk of suicide (Multimedia Appendix 1 provides the trigger words). We also kept observation notes about the provider responses.

#### Qualitative Feedback

We collected qualitative feedback in several ways. We tracked responses to our monthly SMS text message prompts to participants asking, “What is the most positive part of receiving these text messages?” and “What do you not like about receiving the text messages?” We also invited all adolescent participants to share feedback in a semistructured interview after their CBT-D completion with an incentive of US $30 Amazon gift card. The interviews were moderated by a member of our clinical research team who followed a semistructured guide containing the study objectives to explain, questions to pose, and prompts to use when needed. Specifically, we explained that researchers hoped to obtain information about mHealth interventions such as HealthySMS and group CBT services for depression. Next, we asked for general feedback about the HealthySMS system and then specifically asked about different aspects of HealthySMS, such as the mood prompts, responses to mood ratings, and group reminders. We also showed participants a list of the HealthySMS texts and asked for feedback on their impact, content, and phrasing. Textbox 1 lists the interview questions and prompts. Each interview lasted between 19 and 45 (mean 32, SD 8.8) minutes, with the length depending on the amount of details provided by the respondents.

**Qualitative semistructured interview questions and prompts.**

**How did you like the LET’S TEXT! message program overall?**

- Was there anything that made it difficult for you to receive the messages? For example, how did you like: the timing of the messages, the amount of messages, the phrasing of messages?
- Was there anything that made it difficult for you to respond to the messages? For example, how did you like: the timing of the messages, the amount of messages, the phrasing of messages?
- Is there anything about it you would suggest we change?

**How did you like the LET’S TEXT! Mood Prompts and Responses? (after general feedback was given, participants were shown the list of printed texts for specific feedback on this question)**

- What did you think worked well?
- What was difficult or did not go well?
- Is there anything you would change?
- Did these messages change your mood, thoughts, or behavior?

**How did you like the LET’S TEXT! Skill Practice Reminders? (after general feedback was given, participants were shown the list of printed texts for specific feedback on this question)**

- What did you think worked well?
- What was difficult or did not go well?
- Is there anything you would change?
- Did these messages change your mood, thoughts, or behavior?
- For example, did you need more/less help with any of the skills; was the purpose of the reminders clear; -were the messages too few/many; were the reminders relevant to your goals; were they phrased appropriately?

**How did you like the LET’S TEXT! Group Reminders?**

- Did these messages change your mood, thoughts, or behavior?

**Some teens find...**

- ...it easier to participate if they feel: comfortable, respected, and understood by the group members and group leader attached to the messages. How was your relationship with the group and group leader and did that affect your experience with the text messages?
- ...that the messages help them become more active. Do you think your activity completion was impacted by the text messages?
- ...the messages help them go to group and participate more often and/or effectively. How was your group engagement and did that change with the text messages?
- ...the messages are reinforcing and help them practice the group skills more often and/or effectively. How was your practice of the group skills and did that change with the text messages?

**Is there ANYTHING ELSE you would like to share that we haven’t asked you?**

### Data Analytic Plan

We analyzed quantitative data on participant characteristics, HealthySMS engagement, and safety keyword triggers using descriptive statistics in SPSS (IBM Corp). We calculated the SMS text message response rates by dividing the number of responses by the number of SMS text messages received for mood ratings.

We analyzed the qualitative data in a multistep process using thematic analysis principles [53]. First, we developed a hierarchical coding system based on recurrent concepts that we uncovered when conducting the qualitative interviews and in consideration of the related theoretical literature. Next, members of our research team reviewed the qualitative message content and interview transcriptions to collaboratively refine recurrent themes while iteratively updating the coding system. Our team selected exemplary quotes for each theme.

## Results

### Hypothesis 1

Our first hypothesis was that most of the adolescents (ie, >75%) invited to HealthySMS would initiate and maintain use. All participants who agreed to the research study (n=17, 100%) opted to enroll in the HealthySMS system and initiate the receipt of messages upon starting CBT-D; 94% (16/17) of the participants maintained use during the CBT-D group experience. Only 1 (6%) participant opted out of the SMS text messages by texting “stop” 30 days after initiation.

### Hypothesis 2

Our second hypothesis was that adolescents enrolled in HealthySMS would display high rates of engagement with the SMS text message system (ie, ≥75% response rate). As shown in Table 2, the HealthySMS response rate varied among participants. The average response rate to daily mood ratings was 61%, with a range of 0.00 to 1.77 responses per message. Of the 17 participants, only 1 (6%) participant did not respond to any of the SMS text messages prompting mood ratings, and 2 (12%) participants responded multiple times to several prompts (indicated by a response proportion >1). Most participants (10/17, 59%) responded to >50% of the daily mood rating prompts.

**Table 2:** HealthySMS engagement.

Participant	Proportion of responses to mood ratings	Weeks until opting out
1	0.04	N/A^a^
2	0.16	N/A
3	0.30	N/A
4	0.64	N/A
5	0.01	N/A
6	1.54	N/A
7	0.00	N/A
8	0.87	N/A
9	0.05	N/A
10	0.82	N/A
11	1.77	N/A
12	0.12	N/A
13	0.87	N/A
14	0.77	N/A
15	0.79	N/A
16	0.75	N/A
17	0.84	4

^a^N/A: not applicable.

### Hypothesis 3

Our third hypothesis was that we would uncover meaningful qualitative themes from participants’ SMS text message responses and semistructured interviews about the feasibility and acceptability of HealthySMS. We posited that meaningful themes would be beneficial in informing iterative system and research protocol improvements supporting future HealthySMS effectiveness research. When examining the context of participants’ SMS text message responses to our monthly message prompts and semistructured interviews asking for feedback, we identified themes and exemplary quotes regarding the feasibility and acceptability of HealthySMS (Tables 3-5).

**Table 3:** Qualitative themes uncovered and example quotes regarding HealthySMS’s feasibility

Theme	Example quote supporting feasibility	Example quote about limited feasibility
Text modality	“I am not really great with the emails. I think just going through texts is almost always better.”	“For a while, I just didn’t even open the texts.”
Consistency of delivery	“I always got the text and sometimes if I didn’t reply, like with my mood, I would get like a reminder, maybe 10 minutes later, respond your mood. So I would say technically or technologically, it was all good. I didn’t run into any issues.”	“Sometimes my mood ratings just wouldn’t come in. Like some days, like towards the ending of it, they just came in periodically like not every day.”
Amount of effort to use	“[It was fine to] like, give a number, how was your day? But if it was like kind of describe your day, I don’t think anyone would want to do that because it would take too long.”	“If you didn’t like type in your mood right away, it would like send you a reminder a lot. So I got those a lot if I wasn’t doing it.”
Timing of texts	“I definitely didn’t get anything like super early or late. I would say they did a pretty good job of, like, changing up time, so it wasn’t like the same time every like 8AM and 10PM, like it was pretty good at switching up times so you could get like different times of the day.”	“I guess sometimes they came at weird times, like really early in the morning or really late at late night...It kind of felt less helpful if they came later in the day because either I had already figured it out or like got past it or it just didn’t help anymore.”
Amount of texts	“I don’t remember getting, like, bombarded with them. So I would say the amount is probably pretty good and reasonable.”	“It felt sometimes like it was getting too many. But that was mainly because that was like at a time when I didn’t need them and so it just felt like a waste of message if that makes sense.”
Text length	“They’re all quick...It’s short and simple and sweet...”	“I liked it, but it was also like kind of a lot sometimes.”

**Table 4:** Qualitative themes uncovered and example quotes regarding HealthySMS’s acceptability

Theme	Example quote supporting acceptability	Example quote about limited acceptability
Group reminders	“I liked it, because sometimes when things got a little hectic, I would forget about group so it’s a useful reminder.”	“They weren't the most helpful, especially because I'm pretty sure I only ever got one text and it was just ‘there is group tomorrow. Don’t forget your binder,’ which I realize is probably works a lot better when you actually have to go somewhere to do it. But like I already said, I have nothing else going on [in quarantine], so I remember yep, that’s tomorrow. I felt like there definitely could be some variation in things like maybe it could bring up, like, remember the skills you learned or don’t be scared to share or something like that, that could be a little more emotional based.”
Personalized response texts	“I did like how if your mood was like a bad mood, it would give you like, it wouldn’t it just be like that sucks feel better, but it would give you like advice and strategies of how you can get better. I think that was nice. And I liked when if you were in a good mood, it would kind of still give you like a different kind of advice to keep you in that space and like be like, like you could use that experience to feel better later when you remember.”	“...having a little bit of background about like who it is I’m responding to, like, is it a computer or is it a person like this data is being used for what, kind of thing might be helpful.”
Mood ratings	“The rating of the mood...it kind of helped me figure out how I was feeling, like in a number form.”	“I would say it was kind of easy when I was like doing super well or super bad..., but little bit harder, I guess, like to be: ‘OK, well, I don’t really know. I’m average.’”
Skill reminder texts overall	“I thought they really were helpful...a really nice boost, and it was and it would remind me of the other things I had learned that I could also use to feel better.”	“Um if I was able to do something like kind of just the reminder was helpful. But with COVID a lot of the times, it wasn’t applicable.”
Activity skill reminders	“I always think it’s good to like set goals for yourself so you have something to work towards. So it’s almost like a little bit of motivation.”	“...like: ‘do a new activity’...a lot of activities have like kind of gotten harder to do with everything [in the context of the COVID-19 pandemic].”
Social skill reminders	“I liked the people [texts], because I struggle with my relationships with people.”	“Sometimes I couldn’t hang out with friends. So then sometimes that even made me, like, a little frustrated.”
Cognition skill reminders	“I think sometimes like I get so stuck in like the past or just like in the moment that it’s good to just like think about your future and like the good things that are to come.”	“...it was just odd getting text telling me to, like, change the way I’m thinking because I know like the way my brain works, it’s not going to just happen...So it was just annoying because I would like to make [the thoughts] go away, but they’re not going to.”
Statements vs questions	“I felt that they were good, I like them more when they were more statement based...I felt that just a clear like statement or like advice boost helped more for me personally.”	“I got confused a bit with the questions they’d ask because I wasn’t sure if I was supposed to, like, respond to them and it would respond back or it was just kind of a moment to reflect.”

**Table 5:** Qualitative themes uncovered and example quotes regarding HealthySMS’s potential impact on evidence-based intervention (EBI) engagement.

Theme	Example quote supporting impact on EBI engagement	Example quote about limited impact on EBI engagement
Participation in group sessions	“It was just like a reminder to like start thinking about group. So like before group, I’d just like start thinking about it so I’d like have more to say.”	“I wouldn’t really say that the text messages changed my experience in the actual group. It felt more like kind of a recap throughout the week, and less than, sort of a second part of group, if that makes sense. Yeah like we talked about the text messages a bit in group, but the text messages were always about the thing that happened last week, and so we’d want to move on. It could be helpful if the text messages like cover, like some newer things to introduce you, but um yeah.”
Group homework completion	“That was kind of like a good check in where I was like, OK, did I have homework? What was it? Could that help me? So I would keep that in there.”	“I would definitely do techniques more if I got a text message reminding me to do it [the specific homework rather than the general skill reminder] in the week.”
Connection with others in the group	“It felt like we were closer because we were all getting the same messages and we were all in the same boat together.”	“My experience with my group and group leader was pretty good, but I wouldn’t really say my experience in group and my experience with the text messages were linked.”
Validation and support	“And it felt like someone was like caring about me.”	“But it also could backfire on if you don’t do it [the suggestion in the text], feeling guilty.”
Impact on mood	“It kind of gave me some positivity boost, and it was nice to have someone or something to talk to me while I was feeling that way.”	“I wasn’t doing anything because I couldn’t go out and it just made me think about it more...So it just made me a little bit more sad.”
Behavior change and skill use	“I got something like the, call or spend time with people who make you feel happy...And I ended up calling one of my closest friends and she did bring up my mood a little bit...”	“I didn’t usually act on it, probably just because it was like, OK, well, right now I’m doing something. So then I wouldn’t really remember to do it later, but I can see how, what the idea was.”

### Hypothesis 4

Our final hypothesis was that we would uncover meaningful qualitative themes from participants’ SMS text message responses and semistructured interviews about the potential impact of HealthySMS on EBI engagement. We posited that meaningful themes would be beneficial in informing decisions on which mechanisms of change to evaluate in future HealthySMS effectiveness research. When examining the context of participants’ SMS text message responses to our monthly message prompts and semistructured interviews asking for feedback, we identified themes and exemplary quotes regarding the potential impact of HealthySMS on EBI engagement (Table 5).

### Safety During the HealthySMS Intervention

A secondary aim of our pilot study was to monitor the HealthySMS safety keyword alert triggers and provider responses to inform system and research protocol adjustments before future HealthySMS research. During the study period, 76 (7.58%) of the 1002 total SMS text messages sent by participants alerted providers to potential suicide risk throughout our flagged keyword system. When examining the content of participants’ SMS text message responses that were flagged for risk of suicide, only 2 (3%) of these 76 messages were determined to contain true risk-related content (eg, texts about wanting to hurt oneself or die by suicide). In one case, the notified provider determined that the text may be an indication that the participant was about to self-harm, and they followed the clinic’s safety protocol; no indication that harm occurred was received, and the participant continued attending the group sessions and engaging in the study. In the second case, the notified provider determined that the text did not represent an increase in risk and addressed the client’s worry about the future in their following session.

## Discussion

### Principal Findings

The results of this feasibility pilot study demonstrate that the use of HealthySMS adjunct to adolescent group CBT depression services (CBT-D) appears feasible and acceptable, as evidenced by high rates of HealthySMS initiation and low rates of dropout, as well as meaningful themes uncovered from participants’ qualitative feedback. Importantly, the findings also provide evidence regarding iterative improvements to the HealthySMS system and research protocol, as well as potential mechanisms of change for enhanced EBI engagement and, ultimately, adolescent depression outcomes, which can be used in future effectiveness research. It is compelling that the results of this study were obtained in the context of ongoing clinical services at a real-world outpatient clinic experiencing a transition to telehealth services amidst the onset of the COVID-19 pandemic and that the research protocol did not alter the clinical service procedures in any way, such as by incentivizing adolescents to attend the group sessions. Furthermore, it should be noted that we were able to implement this intervention in a safe manner during at a time when an increasing number of youths were at a risk for suicide; the 2 instances of HealthySMS alerts indicating risk were managed via clinical procedures, and no adverse outcomes occurred to any participant during the study.

### HealthySMS Feasibility and Acceptability

As predicted, adolescents enrolled in and maintained the use of HealthySMS at high rates; in fact, no adolescent who agreed to the research study declined initiation of HealthySMS, and only 1 (6%) of the 17 enrolled adolescents opted out after a month of use. HealthySMS response rate was slightly lower than predicted but, of note, varied among participants. Some adolescents had very low response rates (ie, n=1, 6% never responded to any messages), and some adolescents had high response rates (ie, n=1, 6% responded multiple times to most prompts). Overall, most adolescents responded more than half of the time to daily mood rating prompts (ie, response rates averaging >60%). Qualitative feedback suggested that quick and short messages, as well as midday versus early or late message timing, may be the most feasible to respond to. In addition, some adolescents shared that they were motivated to respond by the “interactive” nature of the mood rating texts, which triggered responses 20% of the time. We received feedback that the number of HealthySMS messages and reminders felt appropriate for some participants; however, we received other feedback indicating a preference for less frequent messages and reminders. A future direction to explore is whether personalizing the message and reminder timing and frequency (eg, by requesting participants to share their preferences in the initial survey and monthly feedback prompts to adapt the system to allow for differences by preference) may increase response rates. Interestingly, some adolescents also sent SMS text messages back to the skill reminder messages (ie, response rates averaging 12%), although there were no explicit requests for responses. Adding explicit response requests to skill reminder messages may be another way to explore the potential benefits of HealthySMS.

Regarding the HealthySMS safety triggers, the system appeared to appropriately monitor risk. Throughout the 10 months of the study, 76 (7.58%) of the 1002 total messages sent by participants during the study period triggered provider alerts via flagged keywords (eg, cut, kill, and die; listed in Multimedia Appendix 1). We added words to the system for the current trial after consulting with data from the adolescent crisis text line. Importantly, only 2 of the alerts in this study indicated a potential suicide risk and required clinical follow-up. Providers complied with the established clinical policies and procedures, and no known adverse outcomes occurred among participants throughout the study. In fact, the vast majority of alerts were false positives (eg, “I just got my haircut,” “I went for a bikeride over the bridge,” and “my throat hurts”). One of the most sensitive keywords appeared to be “end,” given that it is a frequently used word and is included in many words (eg, “almost the end of the day” and “FaceTime with friend”). This information can be used to iteratively improve the HealthySMS system to balance the need to detect safety concerns with high sensitivity while trying to avoid false positives and, therefore, reducing providers’ burden in reviewing the alerted SMS text messages, such as by programming keyword alerts to trigger only when the word “end” by itself is sent and not when it is included in other words, such as “friend.” It will be important for future HealthySMS efforts to consider how future HealthySMS research designed to evaluate the appropriateness of the safety keyword triggers and subsequent provider responses to prevent self-harming behaviors would be beneficial.

### Potential Impact on EBI Engagement to Be Evaluated in Future Effectiveness Research

When asked about the utility of HealthySMS, we received input providing initial evidence that the system may indeed enhance EBI engagement. Our identified themes may be used to inform decisions on which mechanisms of change to evaluate in future HealthySMS effectiveness research designed to measure the impact of the system on EBI engagement and, ultimately, adolescent depression outcomes. To begin, most adolescents gave feedback that group reminder texts may have increased their likelihood to attend and meaningfully participate in the group sessions, although one of the participants felt that the group reminder texts were intrusive and did not impact engagement in the group sessions. Variation in responses again highlighted that a personalized SMS text messaging system would be ideal for accommodating individual needs and desires.

Qualitative responses also suggest that the HealthySMS skill messages provided beneficial reminders and motivation for some adolescents to engage in CBT strategies, including behavioral activation and helpful thinking. Some participants did suggest that we sync skill messages with the content being delivered each week (rather than with each module, as is the current setup) to optimize relevance. Although weekly agendas are generally known from the beginning, given the CBT-D format in this study, group providers may adjust agendas to meet the needs of the current group members. Thus, personalizing the timing and content of messages and syncing messages more precisely to weekly content would likely be perceived as helpful by participants but would need to be weighed against the feasibility of such measures, which could increase providers’ burden.

We were also interested in the impact of HealthySMS on the interpersonal connection of the adolescents in the current trial, given the reports of adults in our team’s prior trial of HealthySMS that participants felt cared for and supported by the messages, as well as closer to the CBT group (sources removed for masked review). We received feedback suggesting that HealthySMS may help some adolescents feel more connected to the group leader and other members, as well as validated and supported even with the knowledge that it is a “bot” responding. One of the providers shared that a participant felt that HealthySMS was like a “friend.” Feedback from participant interviews indicated that this sense of connection was especially true when they received the responses to their mood rating texts, which were aligned with how they rated their mood (ie, different responses were sent for high, medium, or low mood ratings).

### Limitations and Future Directions

Our study has several limitations that should be acknowledged and addressed in future work. First, our pilot study investigating the feasibility and acceptability of HealthySMS in a real-world treatment setting was not designed to evaluate the system’s role in improving the ultimate target of adolescent depression. To accomplish this, future effectiveness research building on the lessons learned and iteratively updated deliverables (ie, the HealthySMS system and research protocol) from this study is warranted. Specifically, although we were able to explore the potential impact of HealthySMS on EBI engagement in participants’ qualitative feedback, we did not have the data or sample size required to quantitatively evaluate this construct. In addition, our decision to conduct this pilot study in a real-world setting without making changes to existing clinical procedures created several potentially confounding variables among participants, such as the number of other EBI services engaged in adjunct to CBT-D. Subsequent studies fully powered to detect mechanisms of change as well as control for potential covariates to EBI engagement and outcomes using a control group are called for. Finally, this pilot study took place in the initial months of SIP enforcement owing to the COVID-19 pandemic; thus, it is not known how well our findings will generalize beyond this context.

### Conclusions

Our pilot study suggests that HealthySMS adjunct to the most evidence-based treatment for adolescent depression (ie, CBT) is feasible and acceptable, warranting future effectiveness research evaluating the system’s impact on adolescent EBI engagement and subsequent depression outcomes. As SMS text messaging is cheap and uses technology already in the hands of most adolescents, it is well suited to be added to existing clinical services. Feedback from the participants in our studies suggested that mHealth may be particularly helpful during times of SIP enforcement, given the limited ability for adolescents to engage in activities, social interactions, in-person mental health treatment, and safety monitoring by adults in their lives (eg, teachers and providers). However, it is possible that SMS text message systems are beneficial adjuncts to EBIs in all contexts, given their potential to increase the likelihood and effectiveness of service participation, enhance feelings of connectedness and validation, and encourage skill use in-between sessions. Continued service research on the implementation and effectiveness of mHealth tools has the potential to improve mental health services for a population experiencing drastic increases in depression and suicide risk: adolescents.

## Appendix

Multimedia Appendix 1 Risk alert words.

## Abbreviations

BRIGHT: Building Recovery by Improving Goals, Habits, and Thoughts
CBT: cognitive behavioral therapy
CBT-D: Cognitive Behavioral Therapy for Depression Group for Adolescents
dHealth: digital health
EBI: evidence-based intervention
eCBT: electronic cognitive behavioral therapy
mHealth: mobile health
SIP: shelter-in-place
UCSF: University of California San Francisco

## References

[1] Costello EJ, Copeland W, Angold A (2011). Trends in psychopathology across the adolescent years: what changes when children become adolescents, and when adolescents become adults?. J Child Psychol Psychiatry.

[2] Lu W (2019). Adolescent depression: national trends, risk factors, and healthcare disparities. Am J Health Behav.

[3] Maughan B, Collishaw S, Stringaris A (2013). Depression in childhood and adolescence. J Can Acad Child Adolesc Psychiatry.

[4] Clayborne ZM, Varin M, Colman I (2019). Systematic review and meta-analysis: adolescent depression and long-term psychosocial outcomes. J Am Acad Child Adolesc Psychiatry.

[5] Copeland WE, Alaie I, Jonsson U, Shanahan L (2021). Associations of childhood and adolescent depression with adult psychiatric and functional outcomes. J Am Acad Child Adolesc Psychiatry.

[6] Weavers B, Heron J, Thapar AK, Stephens A, Lennon J, Bevan Jones R, Eyre O, Anney RJ, Collishaw S, Thapar A, Rice F (2021). The antecedents and outcomes of persistent and remitting adolescent depressive symptom trajectories: a longitudinal, population-based English study. Lancet Psychiatry.

[7] Danese A, Smith P (2020). Debate: recognising and responding to the mental health needs of young people in the era of COVID-19. Child Adolesc Ment Health.

[8] Hill RM, Rufino K, Kurian S, Saxena J, Saxena K, Williams L (2021). Suicide ideation and attempts in a pediatric emergency department before and during COVID-19. Pediatrics.

[9] Liang L, Ren H, Cao R, Hu Y, Qin Z, Li C, Mei S (2020). The effect of COVID-19 on youth mental health. Psychiatr Q.

[10] Power E, Hughes S, Cotter D, Cannon M (2020). Youth mental health in the time of COVID-19. Ir J Psychol Med.

[11] Davenport TA, Cheng VW, Iorfino F, Hamilton B, Castaldi E, Burton A, Scott EM, Hickie IB (2020). Flip the clinic: a digital health approach to youth mental health service delivery during the COVID-19 pandemic and beyond. JMIR Ment Health.

[12] Kannarkat JT, Smith NN, McLeod-Bryant SA (2020). Mobilization of telepsychiatry in response to COVID-19-moving toward 21 century access to care. Adm Policy Ment Health.

[13] Oblath R, Twohy E, Higdon C, Duncan A, Folk JB, Schiel MA, Grewal S, Hawks JL, Martinez W, Coble K, Edwards S, Goetz A, Ramtekkar U, Kulkarni CA, Khan S, Doan BT, Nallapula K, Fornari VM, Fortuna LR, Myers K (2023). The provision and utilization of telehealth within academic mental health clinics in North America during the COVID-19 pandemic. JAACAP Open.

[14] Tolou-Shams M, Folk J, Stuart B, Mangurian C, Fortuna L (2022). Rapid creation of child telemental health services during COVID-19 to promote continued care for underserved children and families. Psychol Serv.

[15] Fortuna LR (2022). Editorial: disparities in access to child psychiatric services: can we shift the landscape?. J Am Acad Child Adolesc Psychiatry.

[16] Islam S, Sanchez AL, McDermott CL, Clapp D, Worley J, Becker-Haimes EM (2023). To proceed via telehealth or not? Considerations for pediatric anxiety and related disorders beyond COVID-19. Cogn Behav Pract (Forthcoming).

[17] Curry JF, Meyer AE, Steele RG, Roberts MC (2020). Evidence-based psychosocial treatments of depression in adolescents. Handbook of Evidence-Based Therapies for Children and Adolescents: Bridging Science and Practice.

[18] Klein Jesse B, Jacobs Rachel H, Reinecke Mark A (2007). Cognitive-behavioral therapy for adolescent depression: a meta-analytic investigation of changes in effect-size estimates. J Am Acad Child Adolesc Psychiatry.

[19] Maalouf FT, Brent DA (2012). Child and adolescent depression intervention overview: what works, for whom and how well?. Child Adolesc Psychiatr Clin N Am.

[20] Martin F, Oliver T (2019). Behavioral activation for children and adolescents: a systematic review of progress and promise. Eur Child Adolesc Psychiatry.

[21] Hu T, Zhang D, Yang Z (2015). The relationship between attributional style for negative outcomes and depression: a meta-analysis. J Soc Clin Psychol.

[22] Kanter JW, Manos RC, Bowe WM, Baruch DE, Busch AM, Rusch LC (2010). What is behavioral activation? A review of the empirical literature. Clin Psychol Rev.

[23] Keles S, Idsoe T (2018). A meta-analysis of group Cognitive Behavioral Therapy (CBT) interventions for adolescents with depression. J Adolesc.

[24] Rosselló J, Bernal G, Rivera-Medina C (2008). Individual and group CBT and IPT for Puerto Rican adolescents with depressive symptoms. Cultur Divers Ethnic Minor Psychol.

[25] Luo C, Sanger N, Singhal N, Pattrick K, Shams I, Shahid H, Hoang P, Schmidt J, Lee J, Haber S, Puckering M, Buchanan N, Lee P, Ng K, Sun S, Kheyson S, Chung DC, Sanger S, Thabane L, Samaan Z (2020). Addendum to ‘a comparison of electronically-delivered and face to face cognitive behavioural therapies in depressive disorders: a systematic review and meta-analysis’ [EClinicalMedicine 24 (2020) 100442]. EClinicalMedicine.

[26] Grist R, Croker A, Denne M, Stallard P (2019). Technology delivered interventions for depression and anxiety in children and adolescents: a systematic review and meta-analysis. Clin Child Fam Psychol Rev.

[27] Lamb T, Pachana NA, Dissanayaka N (2019). Update of recent literature on remotely delivered psychotherapy interventions for anxiety and depression. Telemed J E Health.

[28] Stasiak K, Fleming T, Lucassen MF, Shepherd MJ, Whittaker R, Merry SN (2016). Computer-based and online therapy for depression and anxiety in children and adolescents. J Child Adolesc Psychopharmacol.

[29] Ebert DD, Zarski A, Christensen H, Stikkelbroek Y, Cuijpers P, Berking M, Riper H (2015). Internet and computer-based cognitive behavioral therapy for anxiety and depression in youth: a meta-analysis of randomized controlled outcome trials. PLoS One.

[30] Eckshtain D, Kuppens S, Ugueto A, Ng MY, Vaughn-Coaxum R, Corteselli K, Weisz JR (2020). Meta-analysis: 13-year follow-up of psychotherapy effects on youth depression. J Am Acad Child Adolesc Psychiatry.

[31] Kennard BD, Clarke GN, Weersing VR, Asarnow JR, Shamseddeen W, Porta G, Berk M, Hughes JL, Spirito A, Emslie GJ, Keller MB, Wagner KD, Brent DA (2009). Effective components of TORDIA cognitive-behavioral therapy for adolescent depression: preliminary findings. J Consult Clin Psychol.

[32] Fernandez E, Salem D, Swift JK, Ramtahal N (2015). Meta-analysis of dropout from cognitive behavioral therapy: magnitude, timing, and moderators. J Consult Clin Psychol.

[33] Clarke AM, Kuosmanen T, Barry MM (2015). A systematic review of online youth mental health promotion and prevention interventions. J Youth Adolesc.

[34] Lenhart A (2015). Teens, social media and technology overview 2015. Pew Research Center.

[35] Kauer SD, Reid SC, Sanci L, Patton GC (2009). Investigating the utility of mobile phones for collecting data about adolescent alcohol use and related mood, stress and coping behaviours: lessons and recommendations. Drug Alcohol Rev.

[36] Reid SC, Kauer SD, Dudgeon P, Sanci LA, Shrier LA, Patton GC (2009). A mobile phone program to track young people's experiences of mood, stress and coping. Development and testing of the mobiletype program. Soc Psychiatry Psychiatr Epidemiol.

[37] Silk JS, Steinberg L, Morris AS (2003). Adolescents' emotion regulation in daily life: links to depressive symptoms and problem behavior. Child Dev.

[38] Anderson M, Kumar M Digital divide persists even as lower-income Americans make gains in tech adoption. Pew Research Center.

[39] Bohleber L, Crameri A, Eich-Stierli B, Telesko R, von Wyl A (2016). Can we foster a culture of peer support and promote mental health in adolescence using a web-based app? A control group study. JMIR Ment Health.

[40] Aguilera A, Hernandez-Ramos R, Haro-Ramos AY, Boone CE, Luo TC, Xu J, Chakraborty B, Karr C, Darrow S, Figueroa CA (2021). A text messaging intervention (staywell at home) to counteract depression and anxiety during COVID-19 social distancing: pre-post study. JMIR Ment Health.

[41] Bendtsen M, Müssener U, Linderoth C, Thomas K (2020). A mobile health intervention for mental health promotion among university students: randomized controlled trial. JMIR Mhealth Uhealth.

[42] MacDougall S, Jerrott S, Clark S, Campbell LA, Murphy A, Wozney L (2021). Text message interventions in adolescent mental health and addiction services: scoping review. JMIR Ment Health.

[43] Cole-Lewis H, Kershaw T (2010). Text messaging as a tool for behavior change in disease prevention and management. Epidemiol Rev.

[44] Rempel GR, Ballantyne RT, Magill-Evans J, Nicholas DB, Mackie AS (2014). Texting teens in transition: the use of text messages in clinical intervention research. JMIR Mhealth Uhealth.

[45] Whittaker R (2012). Issues in mHealth: findings from key informant interviews. J Med Internet Res.

[46] Whittaker R, Stasiak K, McDowell H, Doherty I, Shepherd M, Chua S, Dorey E, Parag V, Ameratunga S, Rodgers A, Merry S (2017). MEMO: an mHealth intervention to prevent the onset of depression in adolescents: a double-blind, randomised, placebo-controlled trial. J Child Psychol Psychiatry.

[47] Kobak KA, Mundt JC, Kennard B (2016). Erratum to: integrating technology into cognitive behavior therapy for adolescent depression: a pilot study. Ann Gen Psychiatry.

[48] Aguilera A, Bruehlman-Senecal E, Demasi O, Avila P (2017). Automated text messaging as an adjunct to cognitive behavioral therapy for depression: a clinical trial. J Med Internet Res.

[49] Bruehlman-Senecal E, Aguilera A, Schueller SM (2017). Mobile phone-based mood ratings prospectively predict psychotherapy attendance. Behav Ther.

[50] Craig P, Dieppe P, Macintyre S, Michie S, Nazareth I, Petticrew M, Medical Research Council Guidance (2008). Developing and evaluating complex interventions: the new Medical Research Council guidance. BMJ.

[51] Skivington K, Matthews L, Simpson SA, Craig P, Baird J, Blazeby JM, Boyd KA, Craig N, French DP, McIntosh E, Petticrew M, Rycroft-Malone J, White M, Moore L (2021). A new framework for developing and evaluating complex interventions: update of Medical Research Council guidance. BMJ.

[52] Miranda J, Woo S, Lagomasino I, Hepner K, Wiseman S, Muñoz R (2006). BRIGHT CBT depression group manuals. i4Health, Palo Alto University.

[53] Creswell JW, Clark VL (2007). Designing and Conducting Mixed Methods Research.

