# Supplementary material for: HealthySMS Text Messaging System Adjunct to Adolescent Group Cognitive Behavioral Therapy in the Context of COVID-19 (Let’s Text!): Pilot Feasibility and Acceptability Study
Source: JMIR Ment Health. 2024 Feb 19;11:e49317. doi: 10.2196/49317 (PMC10912989; doi:10.2196/49317)
Supplement: Multimedia Appendix 1 [file mental_v11i1e49317_app1.docx]

**Appendix A: Risk Alert Words**

Kill, Die, Suicide, Suicidal, Don’t want to live, Do not want, Bridge, What is the point, Harm, hang, Hurt, Gun, Cut, Pills, Death, cop, midnight, train, syringe, excedrin, myself, die, antifreeze, noose, revolver, tablets, 11:11, 800mg, bathtub, safe, rum, ibuprofen, looney, nightstand, electrocution, vampire, railroad, bridge, acetaminophen, god, abuse, end, finish, Jesus, dead, heaven, hell, knife, razor, Do not want to live, Don’t want, Hopeless
